# Highly sensitive active pixel image sensor array driven by large-area bilayer MoS_2_ transistor circuitry

**DOI:** 10.1038/s41467-021-23711-x

**Published:** 2021-06-11

**Authors:** Seongin Hong, Nicolò Zagni, Sooho Choo, Na Liu, Seungho Baek, Arindam Bala, Hocheon Yoo, Byung Ha Kang, Hyun Jae Kim, Hyung Joong Yun, Muhammad Ashraful Alam, Sunkook Kim

**Affiliations:** 1 School of Advanced Materials Science and Engineering, Sungkyunkwan University, Suwon, Republic of Korea; 2 Department of Engineering “Enzo Ferrari” (DIEF), University of Modena and Reggio Emilia, Modena, Italy; 3 Department of Electronic Engineering, Gachon University, Seongnam, Republic of Korea; 4 School of Electrical and Electronic Engineering, Yonsei University, Seoul, Republic of Korea; 5 Research Center for Materials Analysis, Korea Basic Science Institute (KBSI), Daejeon, Republic of Korea; 6 School of Electrical and Computer Engineering, Purdue University, West Lafayette, Indiana USA; 7 Department of Electrical and Computer Engineering, The University of Texas at Austin, Austin, Texas 78758 USA

**Keywords:** Electrical and electronic engineering, Nanoscale devices

## Abstract

Various large-area growth methods for two-dimensional transition metal dichalcogenides have been developed recently for future electronic and photonic applications. However, they have not yet been employed for synthesizing active pixel image sensors. Here, we report on an active pixel image sensor array with a bilayer MoS_2_ film prepared via a two-step large-area growth method. The active pixel of image sensor is composed of 2D MoS_2_ switching transistors and 2D MoS_2_ phototransistors. The maximum photoresponsivity (*R*_ph_) of the bilayer MoS_2_ phototransistors in an 8 × 8 active pixel image sensor array is statistically measured as high as 119.16 A W^−1^. With the aid of computational modeling, we find that the main mechanism for the high *R*_ph_ of the bilayer MoS_2_ phototransistor is a photo-gating effect by the holes trapped at subgap states. The image-sensing characteristics of the bilayer MoS_2_ active pixel image sensor array are successfully investigated using light stencil projection.

## Abstract (web summary)

Here, the authors report the realization of an active pixel image sensor array composed by 64 pairs of switching transistors and phototransistors, based on wafer-scale bilayer MoS_2_. The device exhibits sensitive photoresponse under RGB light illumination, showing the potential of 2D MoS_2_ for image sensing applications.

## Introduction

Two-dimensional (2D) transition metal dichalcogenides (TMDs) such as molybdenum disulfide (MoS_2_), molybdenum diselenide (MoSe_2_), tungsten disulfide (WS_2_), and tungsten diselenide (WSe_2_) have been extensively studied as next-generation semiconducting materials due to their attractive electrical and optical properties^1–9^. However, although the TMD flakes obtained via mechanical exfoliation exhibit unique properties, their use in large-scale practical applications is difficult due to their low reproducibility and large property variations^10–17^. By contrast, various large-area growth methods for 2D TMDs have been developed for future electronic and photonic applications^18–25^. Choi et al.^22^ reported a full-color active-matrix organic light-emitting diode display based on large-area MoS_2_ synthesized via metal–organic chemical vapor deposition (CVD). Zhang et al.^23^ reported inverter arrays based on wafer-scale MoS_2_ synthesized via atomic layer deposition (ALD). Choi et al.^24^ reported a curved single photodetector array based on MoS_2_-graphene synthesized via CVD. Large-scale growth methods for TMDs have been reported, but they have not yet been employed to synthesize active pixel image sensors, which are integrated circuits consisting of photodetectors and active transistors that can detect the incident image light and convert it into digital image signals^26–28^.

In this study, we report on an active pixel image sensor array with a large-area bilayer MoS_2_ film, which was directly synthesized on a SiO_2_/Si substrate via a two-step growth method consisting of MoS_2_ sputtering (first step) and sulfurization (second step) without any transfer process. The circuitry in 8 × 8 active pixel image sensor array consists of switching transistors and phototransistors. The phototransistor used as a photodetector in the active pixel image sensor achieves a remarkably high photoresponsivity (*R*_ph_) and signal-to-noise ratio (SNR). The main mechanism responsible for the high photoresponsivity of the bilayer MoS_2_ phototransistor is the photogating (PG) effect induced by light-generated holes trapped at subgap states. This explanation is supported by spectroscopic analysis and by numerical device simulations. The simulations highlight the correlation between threshold voltage (*V*_th_) shift and high *R*_ph_ when including subgap states near the valence band edge. Moreover, both the 64 switching transistors and the 64 phototransistors based on homogeneous semiconductor (i.e., bilayer MoS_2_) in the 8 × 8 active pixel array are systematically investigated. It is revealed that the 64 individual pixels exhibit desired electrical and optical properties and high uniformity. Finally, we demonstrate the image-sensing characteristics of the active pixel image sensor array using light stencil projection. The proposed active pixel image sensor array can potentially be used for future image-sensing applications, such as ultra-thin image sensors, transparent image sensors, artificial-intelligence photosensors, and selective light-detecting imagers^29–32^.

## Results

### Structure design of bilayer MoS_2_ image sensor array

**Fig. 1:**
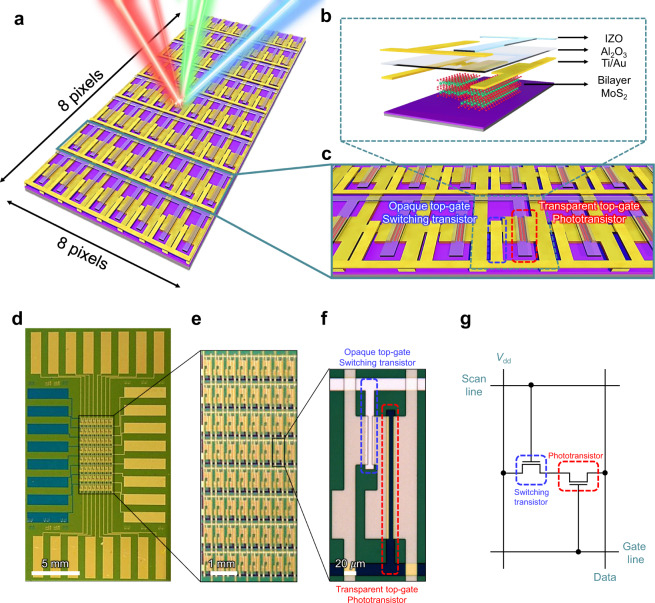
Bilayer MoS_2_ image sensor array. **a** Schematic illustration of an 8 × 8 image sensor array based on bilayer MoS_2_. **b** Cross-section of a pixel consisting of IZO, Al_2_O_3_, Ti/Au, and bilayer MoS_2_. **c** Image sensor array structure design composed of opaque top-gate (Ti/Au electrodes) switching transistor and transparent top-gate (IZO electrodes) phototransistor. **d** Low- and **e** high-magnification photograph of the 8 × 8 image sensor array based on bilayer MoS_2_. **f** Optical microscope image of a pixel composed of opaque top-gate switching transistor (blue-dashed line) and transparent top-gate phototransistor (red-dashed line). **g** A pixel circuit diagram of proposed image sensor array. *V*_dd_ is drain supply voltage.

The active pixel image sensor array with a large-area bilayer MoS_2_ film and its pixel configuration are schematically and photographically illustrated in Fig. 1a–c and Fig. 1d–f, respectively. The designed device is composed of an 8 × 8 pixel array, in which the individual pixels have an opaque top-gate switching transistor and transparent top-gate phototransistor. The opaque top-gate electrode of the switching transistor can completely block the incident light, enabling the pixel selection operation without the influence of the light. By contrast, the transparent top-gate electrode of the phototransistor can successfully transmit the incident light, enabling the generation of electron–hole pairs in the MoS_2_ channel. Figure 1g shows the equivalent circuit of a pixel in the active pixel image sensor array. The phototransistor was integrated into the pixel as a photodetector instead of a photodiode, which is typically used in active pixel image sensors, resulting in a higher photoresponsivity and SNR.

### Fabrication process of bilayer MoS_2_ image sensor array

**Fig. 2:**
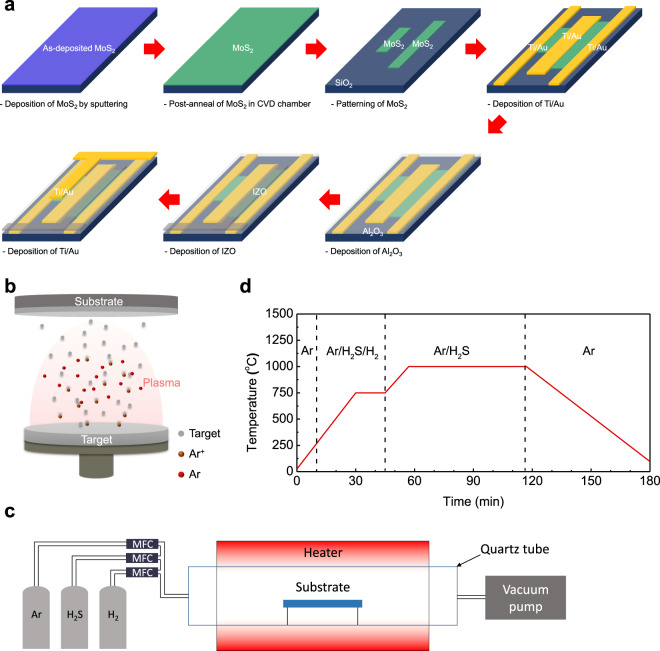
Fabrication steps of the image sensor array based on bilayer MoS_2_ film. **a** Fabrication process of a pixel unit cell in the proposed active pixel image sensor array with a bilayer MoS_2_ film. Synthesis of a bilayer MoS_2_ film using a two-step growth method: MoS_2_ sputtering (first step) and sulfurization in the CVD (second step). Schematic image of **b** RF magnetron sputtering and **c** CVD chamber composed of gas sources (Ar, H_2_S, H_2_), quartz tube, heater, and vacuum pump. MFC is mass flow controller. **d** Temperature profile and gas injection conditions for sulfurization as a function of time. The vertical dashed lines divide four regions with different gas atmospheres.

The presented architecture of the active pixel image sensor array was formed using a thin film process, owing to the atomically thin bilayer MoS_2_ film synthesized directly on a SiO_2_/Si substrate without any transfer process. Figure 2a shows the fabrication process of the pixel unit cell. The bilayer MoS_2_ film was directly synthesized on the cleaned SiO_2_/Si substrate via a two-step growth method. The synthesized bilayer MoS_2_ film was patterned via O_2_ reactive ion etching using a photoresist mask for channel isolation. Titanium/Au (10/50 nm) as the source/drain (S/D) electrodes were deposited and patterned using an electron-beam evaporator and photolithography via a lift-off technique. Aluminium oxide (80 nm) was deposited as the gate insulator via ALD. Subsequently, transparent top-gate electrodes (i.e., indium zinc oxide, IZO) and opaque top-gate electrodes (i.e., Au) were deposited via sputtering and e-beam evaporation, and patterned via photolithography with wet-etching and lift-off techniques, respectively. The details of the device fabrication are presented in the “Methods” section.

### Synthesis of bilayer MoS_2_ film

**Fig. 3:**
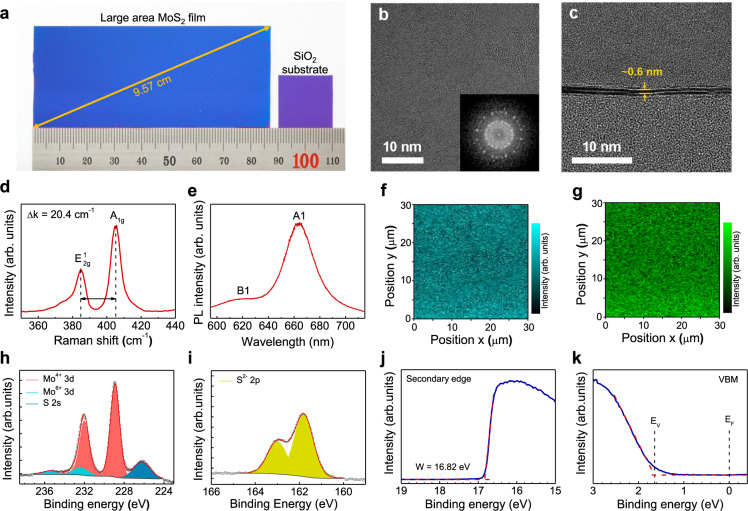
Characterization of large-area bilayer MoS_2_ film. **a** Photograph of a synthesized bilayer MoS_2_ film on SiO_2_/Si substrate (blue) comparison with a bare SiO_2_/Si substrate (purple), indicating the color changes for before and after MoS_2_ growth. **b** Plan-view and **c** cross-sectional TEM images of the bilayer MoS_2_ film. The inset of **b** is a FFT pattern corresponding to the TEM image. **d** Raman and **e** PL spectrum of the bilayer MoS_2_ film. **f**, **g** Raman mapping images of the intensity of *E*^1^_2g_ and *A*_1g_ modes centered at 383 and 404 cm^−1^, respectively. **h**, **i** XPS spectra of Mo 3*d* and S 2*p* core levels of the bilayer MoS_2_ film, respectively. **j** Secondary cut-off and **k** valence band spectra obtained by UPS analysis of bilayer MoS_2_ film.

The two-step growth method consisting of radiofrequency (RF) magnetron sputtering and thermal CVD is schematically illustrated in Fig. 2b, c. A MoS_2_ film was deposited on a SiO_2_/Si substrate via RF magnetron sputtering and then loaded into a CVD chamber for sulfurization. Hydrogen sulfide gas was used as a sulfur precursor in the sulfurization of sputtering deposited MoS_2_, which could cause a homogeneous reaction. The temperature profile and gas injection conditions as a function of time are shown in Fig. 2d. The MoS_2_ film synthesized via the two-step growth method shows a uniform color, indicating thickness uniformity of the MoS_2_ film on SiO_2_/Si substrate with the diagonal length of ~9.57 cm (Fig. 3a).

### Characterization of bilayer MoS_2_ film

High-resolution transmission electron microscopy (TEM) analysis was performed to explore the crystalline structure of the MoS_2_ film. As shown in Fig. 3b, the morphology of the MoS_2_ film appears smooth and consists of several grains. The corresponding fast Fourier transform pattern shows numerous spots forming halo rings, indicating that the MoS_2_ film has several random crystallite orientations^33^. According to Supplementary Fig. 1, the grain size is estimated to be 5–15 nm, indicating a high density of the grain boundaries. The layer number of the MoS_2_ film was determined via the cross-sectional TEM image shown in Fig. 3c. The MoS_2_ film consists of two layers, with an average interlayer spacing of ~0.6 nm, which is consistent with the theoretical and experimental values^34^.

The MoS_2_ film was characterized using Raman spectroscopy to investigate its crystallinity and number of layers. In Fig. 3d, the Raman spectrum shows two bands at 384.8 and 405.2 cm^−1^, which correspond to the in-plane (*E*^1^_2g_) and out-of-plane (*A*_1g_) vibration modes, respectively^35^. As the two modes exhibit a well-defined dependence on the number of layers, the frequency difference (∆*k*) between the two modes is 20.4 cm^−1^, indicating a bilayer MoS_2_ film^36^. The Raman results are consistent with those of mechanically exfoliated MoS_2_, which indicates the high crystallinity of our MoS_2_ film grown via the two-step method. The thickness of the MoS_2_ film was also characterized using atomic force microscopy shown in Supplementary Fig. 2. The film thickness is estimated as ~1.3 nm, corresponding to a typical bilayer MoS_2_^37^. Photoluminescence (PL) measurement was used to investigate the optical quality of the MoS_2_ film. The PL spectrum exhibits two peaks at ~663 and 617 nm, corresponding to the A1 and B1 direct excitonic transitions at 1.97 and 2.11 eV, respectively (Fig. 3e). This indicates that the PL peaks originate from the intrinsic electronic structure of the grown 2H-MoS_2_^38^. Moreover, a randomly selected area of 30 μm × 30 μm was subjected to Raman mapping. Figure 3f, g display the Raman mapping images of the intensity of the *E*^1^_2g_ and *A*_1g_ modes centered at 383 and 404 cm^−1^, respectively. The uniform color contrast demonstrates the high crystallinity and thickness uniformity of our grown MoS_2_ film at a microscale.

The chemical composition of the synthesized bilayer MoS_2_ film was studied using X-ray photoelectron spectroscopy (XPS). In Fig. 3h, the core-level spectrum of Mo *3d* exhibits two strong peaks at 228.9 and 232.0 eV corresponding to Mo^4+^ *3d*_5/2_ and Mo^4+^ *3d*_3/2_ (Mo-S bonding), respectively, and two weak peaks at 232.4 and 235.5 eV corresponding to Mo^6+^ *3d*_5/2_ and Mo^6+^ *3d*_3/2_ (Mo-O bonding), respectively^39,40^. The Mo-O bonding is attributed to MoO_3_, which is contained in the MoS_2_ target. The atomic fraction of Mo^6+^ *3d* contained in the grown bilayer MoS_2_ is ~10.55%. Notably, no additional peak is observed at the low binding energy of ~229.0 eV, which corresponds to metallic 1T-MoS_2_ or metal Mo^41^. This indicates that the sputtered MoS_2_ is fully sulfurized to form 2H-MoS_2_ without a 1T-MoS_2_ component. In addition, Fig. 3i shows two S *2p* peaks at 161.8 and 163.0 eV, corresponding to the doublet of S^2−^ *2p*_3/2_ and S^2−^ *2p*_1/2_, respectively, which further confirms the 2H-MoS_2_ crystal structure. The calculated atomic ratio between S^2−^ *2p* and Mo^4+^ *3d* is ~2.14, indicating a S-rich MoS_2_ with good crystallinity grown via the two-step method.

Ultraviolet photoelectron spectroscopy analysis was used to study the electronic structure of the synthesized MoS_2_ film under ultra-high vacuum using He I as a monochromatic excitation source. The work function (Φ) can be calculated using Φ = *hv* – *W*, where *hv* is the incident photon energy of 21.22 eV (He I) and *W* is the spectral width extracted from the intersection of the slope of the secondary cut-off spectrum with the baseline (Fig. 3j). The measured work function for the MoS_2_ film is 4.40 eV, for which the value is consistent with the other reports^42–45^. In addition, the valence band maximum (VBM) can also be determined from the intersection of the slope of the first state from Fermi energy (*E*_F_ = 0 eV) with the baseline (Fig. 3k). The extracted difference of energy between the *E*_F_ and VBM is 1.65 eV. As previous reports, the few-layer MoS_2_ exhibit a bandgap from 1.29 to 1.9 eV and it could be inferred that the synthesized MoS_2_ film was an *n*-type semiconductor^46^.

### Electrical characteristics of MoS_2_ devices in the array

**Fig. 4:**
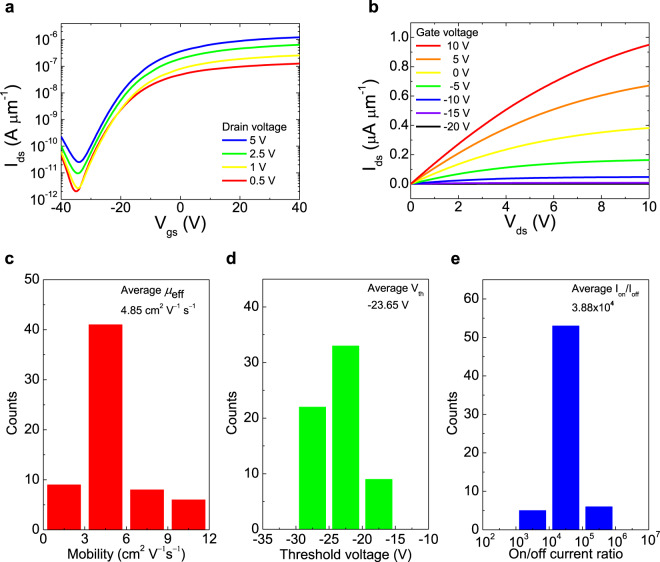
Electrical characteristics and statistical analysis of phototransistors in bilayer MoS_2_ image sensor array. **a** *V*_gs_–*I*_ds_ curves of MoS_2_ phototransistor at *V*_ds_ = 0.5, 1, 2.5, and 5 V. **b** *V*_ds_–*I*_ds_ curves of MoS_2_ phototransistor at *V*_gs_ from −20 to 10 V with the gap of 5 V. Histograms of **c** field-effect mobility (average *μ*_eff_ = 4.85 cm^2^ V^−1^ s^−1^), **d** threshold voltage (average *V*_th_ = −23.65 V), and **e** on/off current ratio (average *I*_on_/*I*_off_ = 3.88 × 10^4^) of the 64 MoS_2_ phototransistors.

*I*–*V* characteristics were measured for the 64 phototransistors and 64 switching transistors in the 8 × 8 active pixel image sensor array, to investigate its electrical properties. Figure 4a shows the transfer curves of a MoS_2_ phototransistor in the active pixel image sensor array, indicating typical *n*-channel behaviors with a current on/off ratio (*I*_on_/*I*_off_) of 5.84 × 10^4^ and a threshold voltage (*V*_th_) of −22.32 V at a drain voltage (*V*_ds_) of 5 V. Figure 4b also shows the output characteristics of the phototransistor. The drain current (*I*_ds_) exhibits a linear behavior at a low drain bias due to the good ohmic contacts between the bilayer MoS_2_ film and the S/D electrodes (Ti/Au), and shows a fully saturated current at a high drain bias due to the velocity-saturated charge carriers. Supplementary Fig. 3 shows comparison with the transfer curves of our bilayer, few-layer, and multilayer MoS_2_ phototransistors under the back-gate and top-gate modulations. Bilayer MoS_2_ exhibits the best performance than the other number of layers of MoS_2_ grown by the two-step method. The electrical properties of MoS_2_ phototransistors show metallic as the number of layer increase over the bilayer. Under back-gate modulation, the bilayer MoS_2_ phototransistor shows improved electrical properties (red line) after Al_2_O_3_ passivation compared with that before Al_2_O_3_ passivation (black line). Under the top-gate modulation, the electrical properties (blue line) of the device were significantly improved compared with that in the case of the back-gate modulation with the Al_2_O_3_ passivation layer (red line), which are attributed to the *n*-type doping effect^47,48^ and the high-*k* dielectric screening effect^49,50^ of the Al_2_O_3_ layer.

The statistical analysis of the electrical performance parameters of the 64 phototransistors in the active pixel image sensor array is summarized in Fig. 4c–e, which was obtained from the transfer curves of the 64 phototransistors as shown in Supplementary Fig. 4a. Figure 4c shows the distribution of the field-effect mobility (*μ*_eff_), which is calculated from the following equation: *μ*_eff_ = ${g}_{\mathrm{m}}\frac{{L}_{{\rm{c}}}}{{W}_{{\rm{c}}}{C}_{\mathrm{ox}}{V}_{\mathrm{ds}}}$, where ${g}_{\mathrm{m}}$ is the transconductance, *L*_c_ and *W*_c_ are the length and width of the channel, respectively, *C*_ox_ is the capacitance of the gate insulator, and *V*_ds_ is the drain voltage. Furthermore, Fig. 4d, e show the distribution of the *V*_th_ and *I*_on_/*I*_off_. All the phototransistors exhibited a highly uniform performance with the following average values: a *μ*_eff_ of 4.85 cm^2^ V^−1^ s^−1^, a *V*_th_ of −23.65 V, and an *I*_on_/*I*_off_ of 3.88 × 10^4^. The 64 switching transistors were quantitatively analyzed using the above method repeatedly. Details of the switching transistors are shown in Supplementary Figs. 4b and 5.

### Photoresponsive characteristics of MoS_2_ devices in the array

**Fig. 5:**
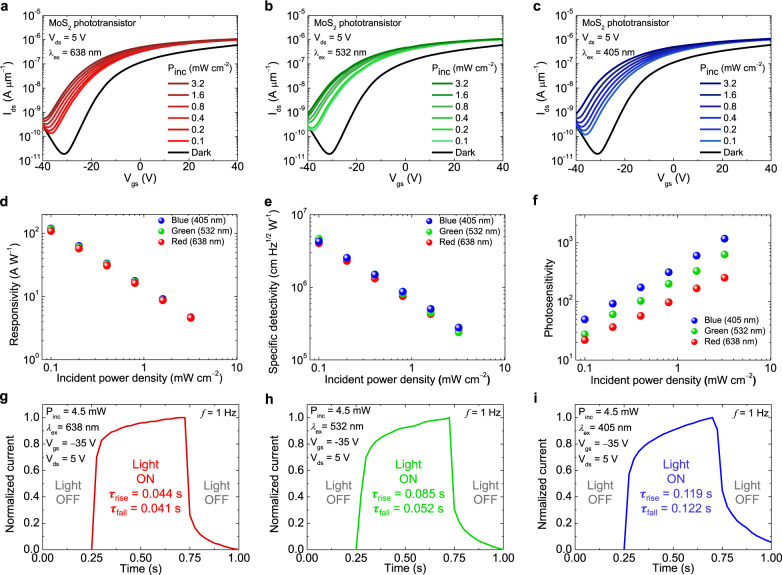
Photoresponsive characteristics of a bilayer MoS_2_ phototransistor in the image sensor array. *I*_ds_–*V*_gs_ curves of a transparent top-gate phototransistor based on bilayer MoS_2_ channel and IZO top-gate at *V*_ds_ = 5 V under **a** red, **b** green, and **c** blue light illumination with various incident power densities (*λ*_ex_ = 638 nm (R), 532 nm (G), and 405 nm (B), and *P*_inc_ = 0.1, 0.2, 0.4, 0.8, 1.6, 3.2 mW cm^−2^). **d** Photoresponsivity, **e** specific detectivity, and **f** photosensitivity of the MoS_2_ phototransistor under R, G, B light illumination calculated from Fig. 4a–c. **g**–**i** Photoswitching characteristics of the MoS_2_ phototransistor under temporal light illumination with *λ*_ex_ = 638, 532, and 405 nm, respectively. All switching curves were measured at *V*_ds_ = 5 V, *V*_gs_ = −35 V, and *P*_inc_ = 4.5 mW cm^−2^ with illumination frequency of 1 Hz. Rise and fall times were extracted from 20% to 80% and from 80% to 20% of the maximum current, respectively.

The proposed transparent top-gate phototransistor structure based on the bilayer MoS_2_ active layer and IZO top-gate electrode in Fig. 1 can easily detect the incident light because of the light transmitted through the transparent top-gate electrode, even using an opaque SiO_2_/Si substrate on which MoS_2_ is directly grown without additional and complex processes (i.e., transferring MoS_2_ onto glass or flexible substrate such as polyethylene terephthalate or polyimide films^22,24,47^, resulting in low productivity and high cost). Figure 5a–c present the photoinduced transfer characteristics of the bilayer MoS_2_ phototransistor in the active pixel image sensor array under red, green, and blue (RGB) light illumination with an excitation wavelength (*λ*_ex_) of 638 nm (R), 532 nm (G), and 405 nm (B) at various incident power densities (*P*_inc_) ranging from 0.1 to 3.2 mW cm^−2^. The photocurrent of the bilayer MoS_2_ phototransistor gradually increased with increasing *P*_inc_. Figure 5d–f show the calculated photoresponsivity (*R*_ph_), specific detectivity (D*), and photosensitivity (*S*_ph_) as functions of excitation wavelength and incident power density, which are important figures of merit for phototransistors. The *R*_ph_ was extracted from the transfer characteristics in Fig. 5a–c, using the equation of *R*_ph_ = *I*_ph_/*P*_inc_ (A W^−1^), where *I*_ph_ and *P*_inc_ are the photocurrent and incident power density, respectively. The D* was obtained by the equation of D* = $\sqrt{A\Delta f}$/NEP = *R*$\sqrt{A\Delta f}$/*i*_n_, where $A$ is the channel area, $\Delta f$ is the electrical bandwidth, NEP is the noise equivalent power, *i*_n_ is the noise current, and *R* is the responsivity at the same measurement conditions as the noise current (*i*_n_)^51^. The noise current, used to obtain the D*, was measured by a lock-in amplifier (Supplementary Fig. 6). We obtained the *S*_ph_ defined as *S*_ph_ = *I*_ph_/*I*_dark_, where *I*_dark_ is the dark current, extracted from the transfer curves of the MoS_2_ phototransistor. The maximum *R*_ph_ of 119.16 A W^−1^ and D* of 4.66 × 10^6^ cm Hz^1/2^ W^−1^ were obtained under the condition of *λ*_ex_ = 405 nm and *P*_inc_ = 0.1 mW cm^−2^ for the former and *λ*_ex_ = 532 nm and *P*_inc_ = 0.1 mW cm^−2^ for the latter. The maximum *S*_ph_ of 1173.44 was obtained at *λ*_ex_ = 405 nm and *P*_inc_ = 3.2 mW cm^−2^. This is high *R*_ph_ reported for phototransistors with large-area synthesized MoS_2_ films as shown in Supplementary Fig. 7 and Table 1.

The main mechanism for the high *R*_ph_ of the bilayer MoS_2_ phototransistor is PG effect by the holes trapped at subgap states^33,52–55^. The TEM and XPS analysis in Fig. 3 show that high density of grain boundaries, excess S, and a small amount of MoO_3_ are present in the grown MoS_2_ film. Previous works report that structural defects in MoS_2_ itself can induce subgap states in the conduction and valence band^56,57^. In Fig. 3k, a narrow distribution of states exists above the VBM in the bandgap. These band tail states near the valence band can capture the photogenerated holes, leading to the accumulation of positive charges and potential barrier lowering (i.e., PG effect). Injecting more electrons from the source by PG effect results in a significant enhancement of photoresponsivity in our MoS_2_ phototransistor. Supplementary Fig. 8 shows the details of PG effect on the bilayer MoS_2_ phototransistor through the extracted *V*_th_ shift ($\triangle$*V*_th_) and *I*_ph_ as a function of *P*_inc_. Supplementary Fig. 8a exhibits $\triangle$*V*_th_–*P*_inc_ curves of the bilayer MoS_2_ phototransistor under RGB light illumination. As depicted in the inset of Supplementary Fig. 8a, holes trapped at subgap states act as a local gate, resulting in the negative shift of *V*_th_ as the *P*_inc_ increased from 0.1 to 3.2 mW cm^−2^. Supplementary Figs. 8b–d show the *I*_ph_–*P*_inc_ curves of the device under RGB light illumination, respectively^53^. By sweeping the gate voltage (*V*_gs_) from −40 to 40 V, the slope of *I*_ph_–*P*_inc_ (i.e., *α*: absorption coefficient) decreases, indicating that the dominant mechanism for *R*_ph_ high switches from photoconductive (PC) effect to PG effect^33,54^. PC effect is that the conductivity of the channel is increased by the photogenerated electron–hole pairs, whereas PG effect is one of special PC effect resulted from accumulation of holes and conduction band lowering due to the defects, impurities, or multi-junction structure of channel materials. The PG effect affects similarly to the additional *V*_gs_, resulting in decrease of α as the *V*_gs_ increases.

Figure 5g–i show the photoswitching behaviors of the bilayer MoS_2_ phototransistor in the active pixel image sensor array under pulsed RGB light illumination with the frequency of 1 Hz. The rise time (*τ*_r_) and fall time (*τ*_f_) were defined as the times taken for the current to change from 20% to 80% and from 80% to 20% of the maximum current, respectively^58^. The photoresponse speeds of the bilayer MoS_2_ phototransistor under RGB light illumination were *τ*_r under R_, *τ*_r under G_, *τ*_r under B_ = 44, 85, 119 ms and *τ*_f under R_, *τ*_f under G_, *τ*_f under B_ = 41, 52, 122 ms, respectively. *τ*_r_ and *τ*_f_ increase as *λ*_ex_ decreases due to the greater number of photogenerated charge carriers. Such a satisfactory photoresponse speed indicates that the bilayer MoS_2_ active pixel image sensor array has the potential for fast image sensing. However, the response time still needs to be improved for high-speed imaging due to the trade-off relationship between response time and responsivity. Therefore, to improve the response speed of our image sensor array, we investigated the photoswitching characteristics of the bilayer MoS_2_ phototransistor with gate pulse as shown in Supplementary Fig. 9. The 40 ms gate voltage pulse significantly enhanced the fall time from 104.8 ms to 22.99 ms due to the detrapping of the trapped holes at subgap states, enabling high-speed operation of the image sensor^29,59–61^. Supplementary Table 2 shows the comparison of the photoresponse speed without and with gate pulse. The bilayer MoS_2_ photodetector without a gate terminal in the same pixel was measured under the same conditions as in Fig. 5 to compare its photoresponsive characteristics with those of the proposed phototransistor (Supplementary Fig. 10). Consequently, compared with the photodetector without a gate terminal, the phototransistor showed significantly improved photoresponsivity, specific detectivity, and photosensitivity (i.e., SNR) by 14.01, 6.68, and 505.79 times, respectively.

### Simulation results of bilayer MoS_2_ phototransistor

**Fig. 6:**
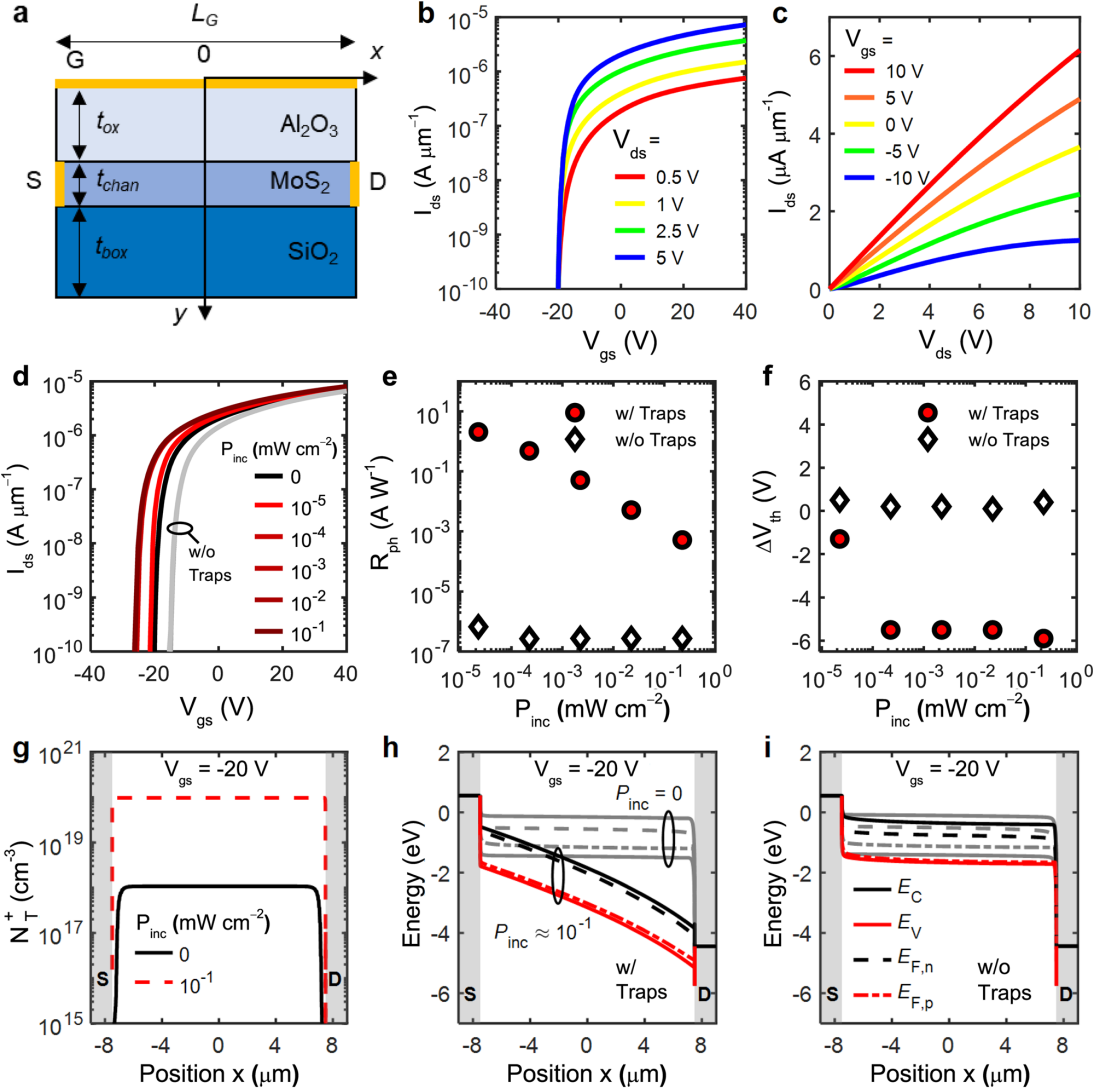
Simulation results. **a** Sketch of the simulated 2D device. *L*_G_, *t*_ox_, *t*_chan_, *t*_box_, S, D, and G are gate length, gate oxide thickness, MoS_2_ channel thickness, buried oxide thickness, source, drain, and gate, respectively. **b**, **c** Simulated *I*_ds_–*V*_gs_ and *I*_ds_–*V*_ds_ curves in the dark. **d** Simulated *I*_ds_–*V*_gs_ under light illumination, for different incident power densities (*P*_inc_). Simulated *I*_ds_–*V*_gs_ curves without traps are also included for comparison (gray lines, shifted to the right for better visibility). **e**, **f** Photoresponsivity (*R*_ph_) and Threshold Voltage Shift (*ΔV*_th_) vs. *P*_inc_ extracted from the *I*_ds_–*V*_gs_ simulation with and without traps, showing the correlation between high *R*_ph_ and negative *ΔV*_th_. **g** Trapped charge density (${N}_{T}^{+}$) at *V*_gs_ = −20 V in the dark (*P*_inc_ = 0 mW cm^−2^, black line) and under light illumination (*P*_inc_ ≈ 10^−1^ mW cm^−2^, red-dashed line). **h**, **i** Simulated energy band diagrams at *V*_gs_ = −20 V in the dark (*P*_inc_ = 0 mW cm^−2^, gray lines) and under light illumination (*P*_inc_ ≈ 10^−1^ mW cm^−2^, black and red lines) with (**h**) and without traps (**i**). *X*-axis in **g**–**i** is the position along the channel from source to drain contacts (indicated by the shaded gray bands).

To support the interpretation regarding the high *R*_ph_ of the bilayer MoS_2_ phototransistor being induced by the PG effect, numerical device simulations were carried out. The simulation framework is based on the drift-diffusion formalism (see “Simulation methods” for details of the simulation setup) with material and device parameters set according to the experimental values derived from Fig. 3. The device structure implemented in the simulator is schematically represented in Fig. 6a (geometrical dimensions and electrical parameters adopted in the simulations are reported in the Supplementary Note 1). The simulated *I*_ds_–*V*_gs_ and *I*_ds_–*V*_ds_ curves in the dark (i.e., with no optically generated carriers) are shown in Fig. 6b, c, respectively. The *V*_th_ of the device under no illumination is ≈ −20 V and was calibrated by assuming that the Fermi energy (*E*_F_) at equilibrium is close to the conduction band edge (*E*_C_). This was simulated by including a large doping density (*N*_D_ = 5 × 10^19^ cm^−3^) in the MoS_2_ channel.

The simulated *I*_ds_–*V*_gs_ curves under different light illumination conditions (each corresponding to a specific incident power density, *P*_inc_) are shown in Fig. 6d. As it can be noticed, simulations correctly anticipate the large photoresponsivity observed experimentally (see Fig. 6e), which is attributed to the negative *V*_th_ shift as shown in Fig. 6f. This behavior was reproduced by including a defect level at 0.2 eV from the valence band edge (*E*_V_), mimicking the narrow distribution of states above the VBM in the bandgap (see Fig. 3k). The trap state is of the donor-like type, i.e., positively charged (neutral) when filled (empty) of holes. As these traps are 0.2 eV above *E*_V_, they tend to become easily filled with holes, the latter being provided by light. When traps capture the optically generated holes, they become positively charged and thus cause *V*_th_ to decrease (Fig. 6f), in turn leading to the high *R*_ph_ (Fig. 6e) as also observed in the experiments (Fig. 5d). Interestingly, when performing simulations without including the trap states, the observed current modulation was relatively weak and inconsistent with experimental results. For example, Fig. 6d shows the simulated *I*_ds_–*V*_gs_ without traps (gray lines, shifted to the right for better readability). In this case, no appreciable *V*_th_ shift is observed as shown in Fig. 6f. This leads to a much lower *R*_ph_, as shown Fig. 6e. Figure 6g shows the trapped charge density (${N}_{T}^{+}$) at *V*_gs_ = −20 V in the dark and with light illumination, clearly indicating that more holes are trapped in the latter case than in the former. Figure 6h,i show the simulated band diagrams corresponding to dark and with light illumination conditions with and without the inclusion of the trap states, respectively. The comparison of the two cases shows the high conductivity modulation occurring in the simulation with the inclusion of traps (Fig. 6h) and the weak variation occurring in the case without traps (Fig. 6i). All these results corroborate the hypothesis of PG effect being responsible for high *R*_ph_.

### Image sensing of bilayer MoS_2_ image sensor array

**Fig. 7:**
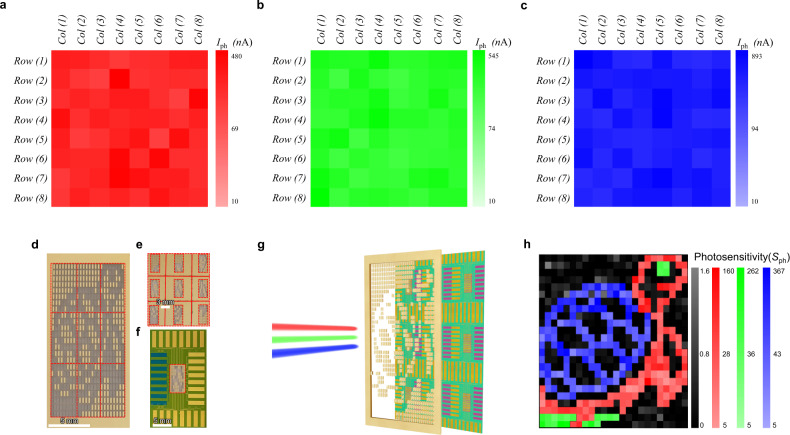
Image-sensing characteristics of 8 × 8 bilayer MoS_2_ image sensor array. **a**–**c** Photocurrent mapping of 64 MoS_2_ phototransistors at *V*_ds_ = 1 V, *V*_gs_ = −10 V under RGB light illumination (*λ*_ex_ = 638, 532, and 405 nm and *P*_inc_ = 3.2 mW cm^−2^), indicating uniform photocurrent photoresponses. **d** Photograph of the designed turtle stencil for projection (total 24 × 24 pixels). **e** Separated turtle stencils for individual light stencil projection on 8 × 8 image sensor array. **f** Top view of the image sensor array covered with a piece of separated turtle stencils. **g** Measurement concept using the light stencil projection for image detection of the image sensor array. The 8 × 8 bilayer MoS_2_ image sensor array is placed behind turtle stencil and measured electrical properties under RGB light illumination (*λ*_ex_ = 638, 532, and 405 nm). **h** Photosensitivity mapping result extracted from image detection of bilayer MoS_2_ image sensor array.

The photoinduced transfer characteristics of all the 64 phototransistors were measured under a light illumination with a *λ*_ex_ of 638, 532, and 405 nm at a fixed *P*_inc_ of 3.2 mW cm^−2^, to investigate the photoresponse uniformity of the active pixel image sensor array. Figure 7a–c show the extracted photocurrent mapping of the 8 × 8 active pixel image sensor array achieved using the photoinduced transfer characteristics of the 64 phototransistors under RGB light illumination. Consequently, it is confirmed that the active pixel image sensor array has a high photoresponse uniformity for all the RGB light illumination conditions. The cross-talk characteristics between adjacent pixels were also investigated, resulting in negligible cross-talk between adjacent pixels (Supplementary Fig. 11). Supplementary Fig. 12 shows the grayscale variation under various incident power densities. Moreover, a designed turtle stencil (total 24 × 24 pixels) was prepared and patterned using a laser cutting system as shown in Fig. 7d and Supplementary Video 1, to investigate the image-sensing characteristics of the 8 × 8 active pixel image sensor array. The stencil separated into nine pieces (Fig. 7e) is sequentially placed on the active pixel image sensor array during light projection (Fig. 7f). The measurement method using the light stencil projection is described in detail in Fig. 7g and the “Methods” section. Figure 7h shows the photosensitivity mapping result of the active pixel image sensor array obtained through the light stencil projection, indicating successful turtle image sensing (the image pixel resolution is 576 pixels). This is due to the unique pixel structure in the active pixel image sensor array composed of opaque top-gate switching transistors and transparent top-gate phototransistors. As the top-gate electrode and block layer, the Au film covers the switching transistor in the pixels to remove the photocurrent interference of the switching transistor by light illumination during the image sensing through the active matrix as shown in Fig. 1. As mentioned earlier, the IZO top-gate electrode of the phototransistors enables light detection by the image sensor due to its transparent property.

Moreover, typical image signal process of complementary metal-oxide-semiconductor image sensor can be simply described as follows. First, the incident light is converted into current by  the image sensor array and then read out by integrated circuit; then, current is converted to a digital signal by analog-to-digital converter. After that, the digital values are transferred into image signal processor for image processing such as image quality improvement. Finally, image data are acquired through the input/output interface^62,63^.

## Discussion

We demonstrated an active pixel image sensor array based on a bilayer MoS_2_ film. A large-area bilayer MoS_2_ film was directly synthesized on a SiO_2_/Si substrate via a two-step growth method without any transfer process. In particular, the active pixel image sensor array architecture comprised opaque top-gate switching transistors and transparent top-gate phototransistors achieved higher photoresponsive characteristics than those of photodetector without gate terminal. The main mechanism for the high *R*_ph_ of the bilayer MoS_2_ phototransistor is PG effect by the holes trapped at subgap states. This was confirmed by detailed, experimentally guided, numerical simulations that highlighted how the large photoresponsivity increase is a consequence of photo-excited hole trapping at subgap states close to the valence band edge. The 64 individual pixels in the 8 × 8 active pixel image sensor array successfully functioned under RGB light illumination. The desired photoresponsive performance and unique architecture of the proposed active pixel image sensor array can facilitate its use in next-generation image detection applications, such as ultra-thin image sensors, transparent image sensors, artificial-intelligence photosensors, and selective light-detecting imagers.

## Methods

### Growth of MoS_2_ via the two-step method

A bilayer MoS_2_ film was synthesized using a two-step method, consisting of RF magnetron sputtering and thermal CVD. *P*-type doped Si substrates covered with 300 nm-thick SiO_2_ were used as the substrates. Prior to the deposition of MoS_2_ via sputtering, the SiO_2_/Si substrates were ultrasonically cleaned for 10 min each in acetone, isopropyl alcohol, and deionized water. A 50.8 mm-diameter MoS_2_ target (99.9%) was used in the magnetron sputtering system. The chamber was maintained below the base pressure of 3 × 10^−6^ Torr after loading the substrates and at the working pressure of 10 mTorr with an Ar flow of 75 s.c.c.m. The MoS_2_ target was pre-sputtered for 10 min before the deposition, to remove the oxide layer on it and to make the plasma stable. The thin MoS_2_ films were deposited on the SiO_2_/Si substrates at room temperature with the RF power of 50 W for 14 s.

The as-deposited MoS_2_ film on the SiO_2_/Si substrate was sulfurized in a 2 inch CVD chamber. The chamber was pumped to a low vacuum, and then Ar was injected with a flow rate of 50 s.c.c.m. For the growth of MoS_2_, the temperature was ramped up to 750 °C in 30 min and maintained for 15 min. A gas mixture of H_2_ (5 s.c.c.m.) and H_2_S (5 s.c.c.m.) was injected into the chamber when the temperature reached 300 °C. Subsequently, an annealing process was performed at 1000 °C for 1 h under an Ar (50 s.c.c.m.) and H_2_S (5 s.c.c.m.) atmosphere. The furnace was rapidly cooled to room temperature under an Ar flow at a rate of 50 s.c.c.m.

### MoS_2_ phototransistor fabrication

The MoS_2_ film was deposited on the SiO_2_/Si substrate for 14 s via RF magnetron sputtering, and then sulfurized at 750 °C and successively annealed at 1000 °C for 1 h in the CVD chamber, to fabricate the active pixel image sensor array with the bilayer MoS_2_ film. Conventional photolithography was used to pattern the MoS_2_ channel. A photoresist was spin-coated for 30 s at 3000 r.p.m. on the MoS_2_ film. Afterward, the coated photoresist layer was exposed to UV light for 1 s and removed with a developer, which covered only the MoS_2_ channel. The uncovered MoS_2_ film was etched with O_2_ plasma at the power of 10 W for 10 s. The photoresist remaining on the MoS_2_ film was removed by spraying acetone. A lift-off resist and the photoresist were sequentially coated using a spin-coater for 45 s at 2000 r.p.m. and 30 s at 3000 r.p.m., respectively. Following that, they were removed via exposure to UV light. Afterward, the MoS_2_ film was subjected to development processes to pattern electrodes. Subsequently, 10 nm titanium and 50 nm gold were deposited as electrodes via electron-beam evaporation and patterned via a lift-off process using photoresist remover. An 80 nm-thick Al_2_O_3_ as the top-gate insulator was then deposited on the lifted-off sample by ALD at 100 °C. Subsequently, IZO was deposited as the top-gate electrodes using a sputtering system. For the chemical etching of the IZO film after patterning, the sample was immersed in buffered oxide etching for 20 s. After patterning the transparent top-gate electrodes, Ti/Au (20/50 nm) were deposited by electron-beam evaporation and pattern by the lift-off process as the opaque top-gate electrodes. Finally, via holes were patterned using the aforementioned conventional photolithography and chemical etching processes sequentially.

### Characterization of MoS_2_

The surface of the MoS_2_ film grown on the SiO_2_/Si substrates was observed using optical microscopy (BX51M, Olympus, Co.) The heights of the bilayer MoS_2_ films were measured using atomic force microscopy (XE7, PSIA Co.) in non-contact mode. Raman and PL spectra were obtained using a micro-Raman spectrometer system (ALPHA300, WITec, Co.) with an excitation laser at 532 nm at the MEMS·Sensor Platform Center of SungKyunKwan University (SKKU). XPS (Theta Probe AR-XPS System, ThermoFisher Scientific) measurements were performed with Al Kα X-ray radiation (1486.6 eV). The working pressure in the ultra-high-vacuum chamber during the measurement was maintained below 3 × 10^−9^ mbar. C 1*s* at 284.5 eV was used for the calibration of the binding energies. The atomic image of MoS_2_ was characterized using TEM (JEM ARM 200F, JEOL). The cross-sectional TEM imaging samples were fabricated using a focused ion beam system (NX2000, HITACHI). The electrical and photoresponse properties of the MoS_2_ active pixel image sensor array were characterized using a semiconductor characterization system (4200 SCS, Keithley) and multi-channel fiber-coupled laser source (MCLS1, Thorlabs) in an ambient condition. The noise current of the MoS_2_ phototransistor was measured using a lock-in amplifier (SR830 lock-in amplifier, Stanford Research Systems). The image-sensing characteristics of the active pixel image sensor array were measured using light stencil projection. The turtle stencil (24 × 24 pixels) was patterned onto the gold-deposited glass using a laser cutting system. The turtle stencil separated into nine pieces (8 × 8 pixels) was sequentially placed on the 8 × 8 active pixel image sensor array. Subsequently, the individual pixels were measured by connecting the contact pads adjacent to the edge of the substrate under a light projection. To obtain the colored image, each pixel of the image sensor array was measured with the turtle stencil under RGB light that differs from pixel to pixel, respectively. The light stencil projection method was inspired and developed from previous reports^30,64^. Lee et al.^30^ reported a single-pixel imager and the image scanning system with three different light sources (RGB).

### Simulation methods

The 2D numerical simulations model the carrier transport problem with the drift-diffusion formalism (see Supplementary Note 1 for the full equation set). This way, Poisson equation and continuity equations are solved self-consistently for each applied bias (in terms of gate-to-source and drain-to-source voltage), to obtain the electrostatic potential and the carriers’ concentration. For simplicity, the light source is considered ideal, i.e., it provides constant electron–hole pair generation rates. Shockley–Read–Hall formalism was adopted to model trap capture and emission dynamics.

## Supplementary information

Supplementary information

Supplementary Video 1

## Data Availability

The data that support the findings of this study are available from the corresponding author upon reasonable request.
