# Supplementary material for: Highly sensitive active pixel image sensor array driven by large-area bilayer MoS2 transistor circuitry
Source: Nat Commun. 2021 Jun 11;12:3559. doi: 10.1038/s41467-021-23711-x (PMC8196169; doi:10.1038/s41467-021-23711-x)
Supplement: Supplementary file 1 — Supplementary information [file 41467_2021_23711_MOESM1_ESM.pdf]

SUPPLEMENTARY INFORMATION for

Highly sensitive active pixel image sensor array driven by large-area bilayer MoS_2 transistor circuitry

Authors

Seongin Hong[†,1], Nicolò Zagni[†,2], Sooho Choo[†,1], Na Liu[†,1], Seungho Baek[1], Arindam Bala[1], Hocheon Yoo[3], Byung Ha Kang[4], Hyun Jae Kim[4], Hyung Joong Yun[5], Muhammad Ashraful Alam[,6] and Sunkook Kim[*,1]*

Affiliations

[1] School of Advanced Materials Science and Engineering, Sungkyunkwan University, Suwon 440-746, Republic of Korea

[2] Department of Engineering "Enzo Ferrari" (DIEF), University of Modena and Reggio Emilia, Modena 41125, Italy

[3] Department of Electronic Engineering, Gachon University, Seognam 13120, Republic of Korea

[4] School of Electrical and Electronic Engineering, Yonsei University, Seoul 03722, Republic of Korea

[5] Research Center for Materials Analysis, Korea Basic Science Institute (KBSI), Daejeon 34133, Republic of Korea.

[6] School of Electrical and Computer Engineering, Purdue University, West Lafayette, Indiana 47907, USA

*Corresponding author. Email: alam@purdue.edu, intel0616@gmail.com

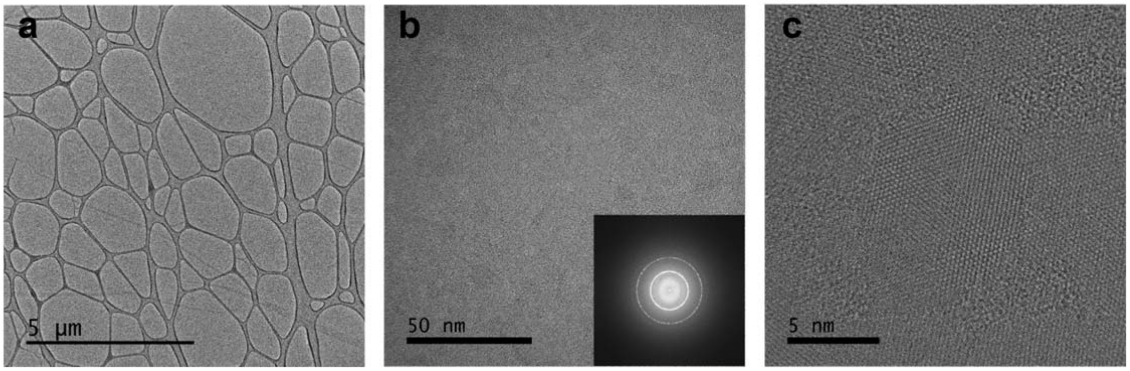

Supplementary Figure 1 | TEM images of bilayer MoS_2 films. a, Low-magnification and **b and c,** high-magnification TEM images. The inset of **b** is a FFT pattern corresponding to the TEM image.

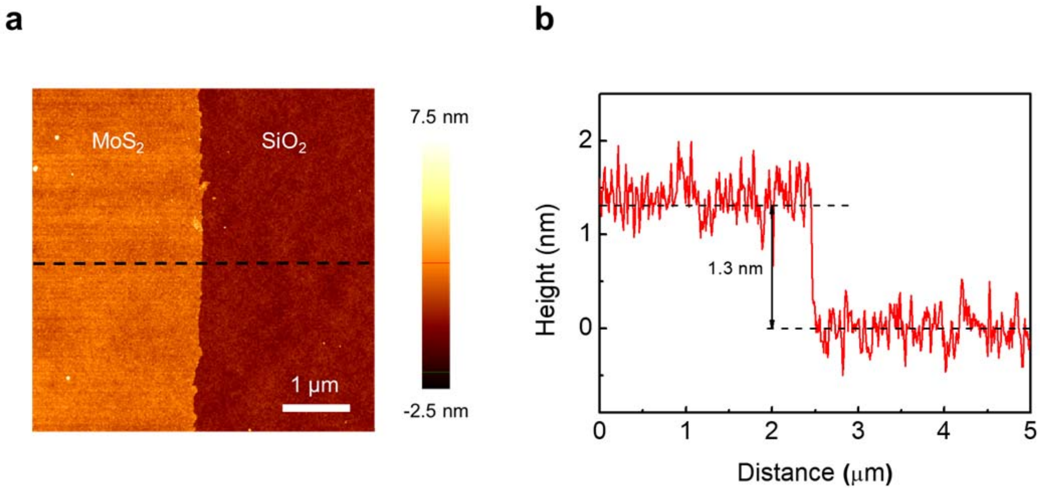

Supplementary Figure 2 I Thickness of a bilayer MoS_2 film directly synthesized on a SiO_2/Si substrate using the two-step growth method. a, AFM image of the bilayer MoS_2 film. **b**. Line profile data of dark-dashed line in the AFM image. The thickness of the MoS_2 film was estimated to be approximately 1.3 nm.

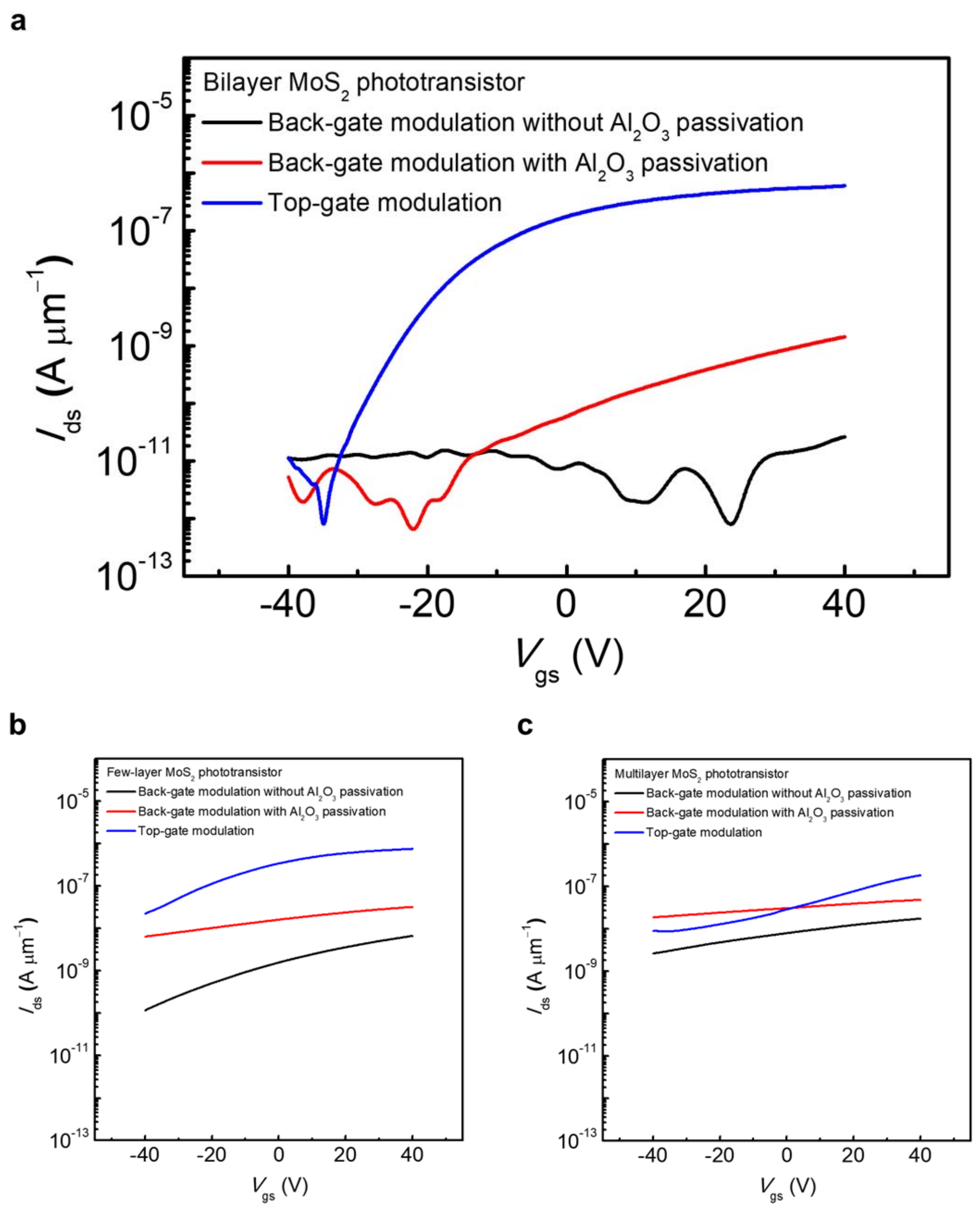

Supplementary Figure 3 l Comparison of the electrical properties of MoS_2 phototransistors. Transfer curves of **a**, bilayer, **b**, few-layer, and **c**, multilayer MoS_2 phototransistor under back-gate modulation without Al_2O_3 passivation (black line), back-gate modulation with Al_2O_3 passivation (red line), and top-gate modulation (blue line).

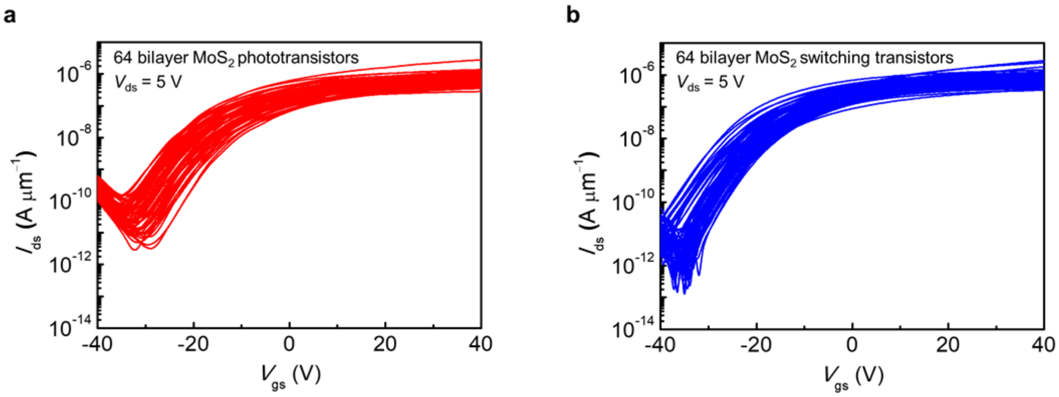

Supplementary Figure 4 l Electrical properties of all the phototransistors and switching transistors in the 8 × 8 active image sensor array with a bilayer MoS_2 film. Transfer characteristics of **a**, 64 phototransistors and **b**, 64 switching transistors at V_{ds} = 5 V.

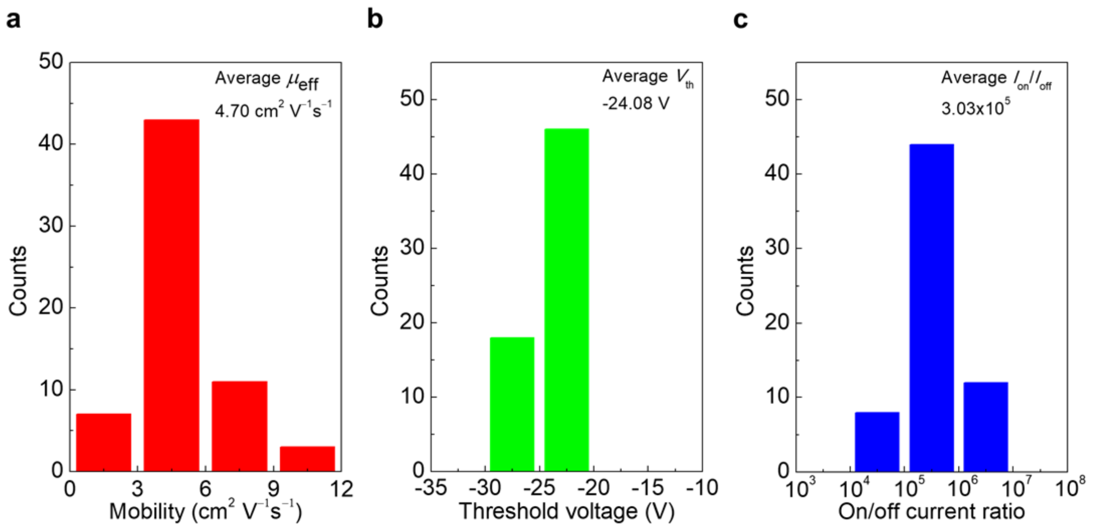

Supplementary Figure 5 l Statistical analysis of switching transistors in the bilayer MoS_2 image sensor array. Histograms of **a**, field effect mobility (average μ_{eff} = 4.70 cm^2 V^{-1} s^{-1}), **b**, threshold voltage (average V_{th} = −24.08 V), and **c**, on/off current ratio (average I_{on}/I_{off} = 3.03 × 10^5) of the 64 MoS_2 switching transistors.

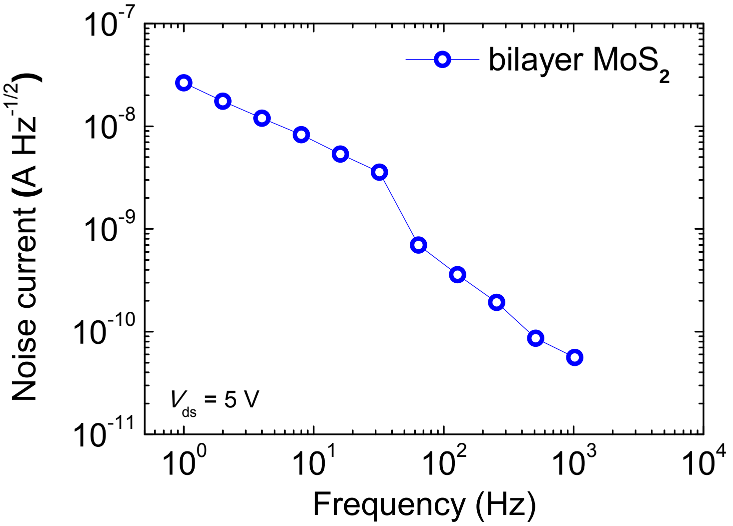

Supplementary Figure 6 l Measured noise current as a function of frequency for the bilayer MoS_2 phototransistors at V_{ds} = 5 V.

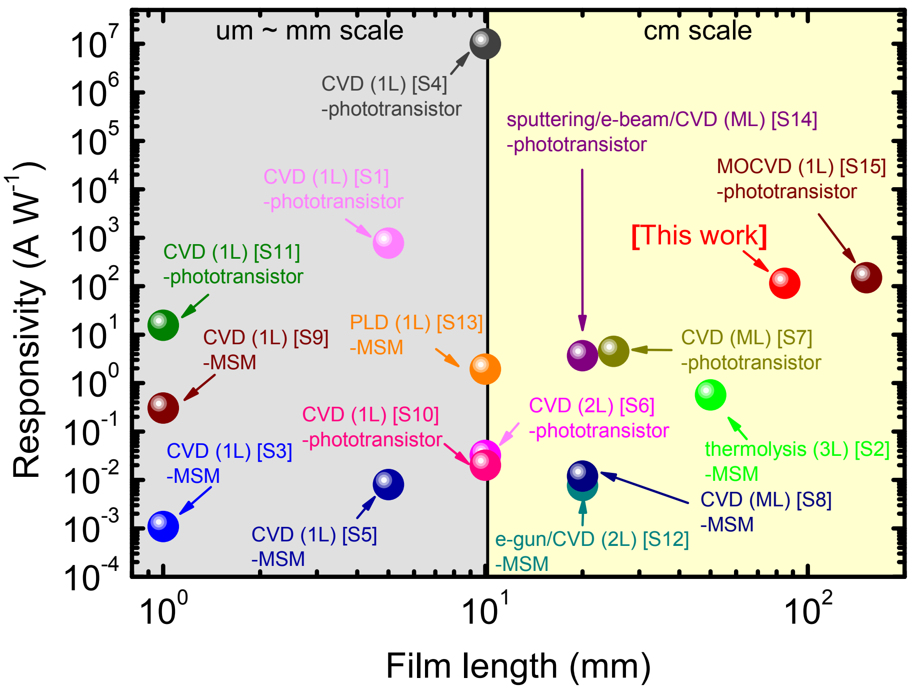

Supplementary Figure 7 I Comparison of the photoresponsivity of the proposed phototransistor based on the synthesized MoS_2 with those of previous studies.

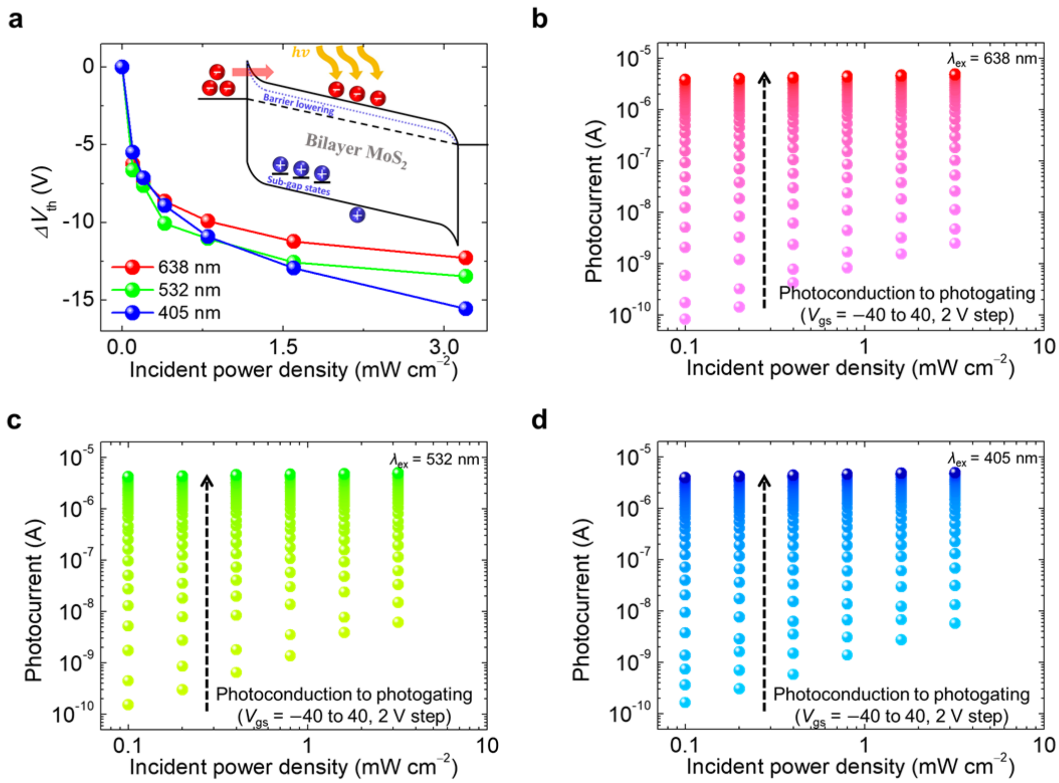

Supplementary Figure 8 l Threshold voltage and photocurrent variation of the bilayer MoS_2 phototransistor as a function of P_{inc} under RGB light illumination. **a**, ΔV_{th}–P_{inc} curves of the MoS_2 phototransistor under RGB light illumination. The inset is the energy band diagram of the MoS_2 phototransistor, indicating the mechanism of the PG effect. I_{ph} - P_{inc} curves of the MoS_2 phototransistor at various V_{gs} under **b**, red **c**, green and **d**, blue light illumination.

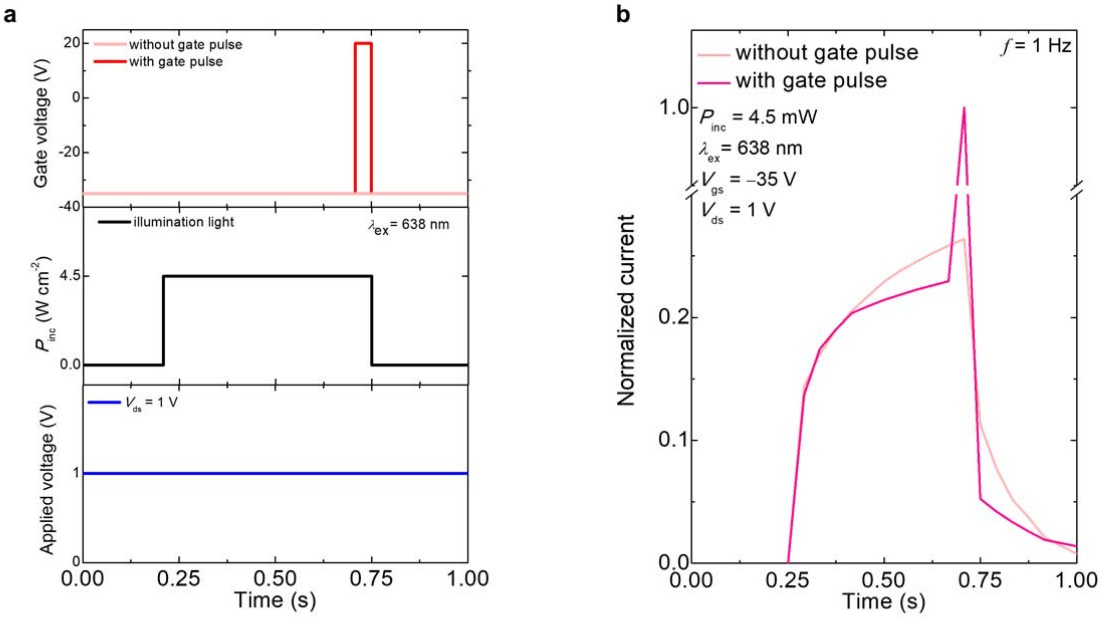

Supplementary Figure 9 l Photoswitching characteristics with gate pulse of a bilayer MoS_2 phototransistor in the image sensor array. a, Measurement conditions for photoswitching properties with gate pulse. Switching curves were measured at V_{ds} = 1 V, V_{gs} = −35 V and P_{inc} = 4.5 mW cm^{-2} with illumination frequency of 1 Hz. The applied gate pulse is 20 V with a width of 40 ms. **b**, Time resolved photoresponsive characteristics of the bilayer MoS_2 phototransistor under temporal light illumination with λ_{ex} = 638 nm without and with gate voltage pulse. The fall time is improved from 104.80 ms to 23.99 ms.

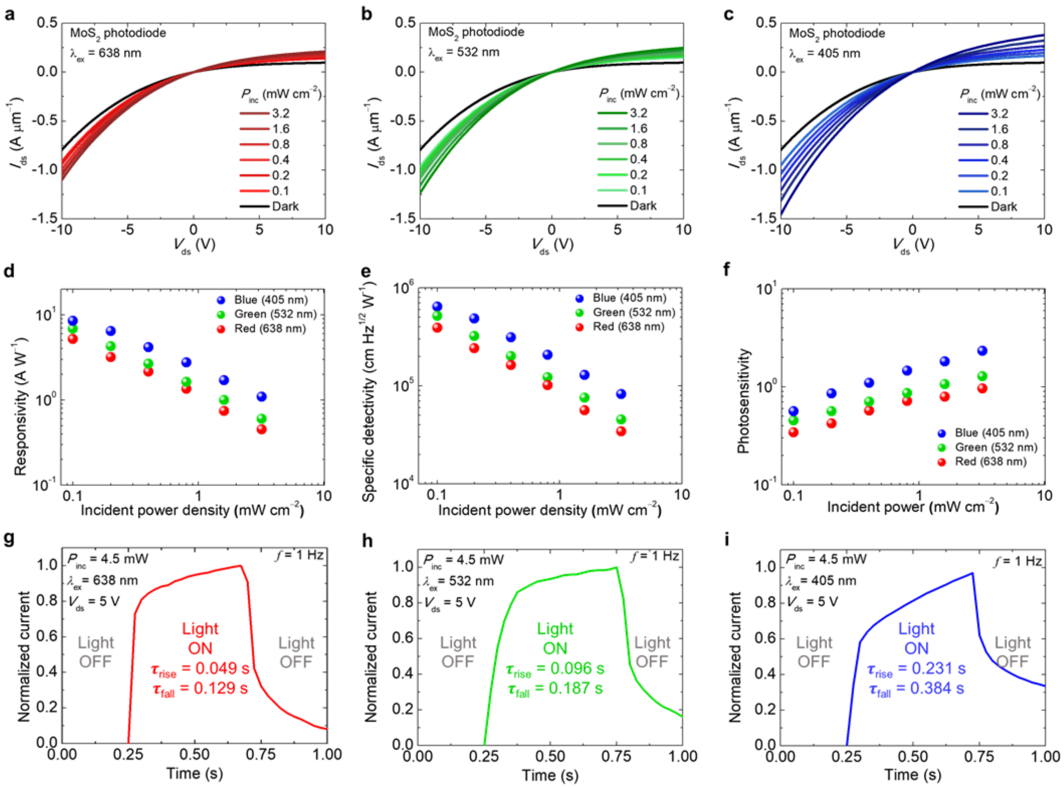

Supplementary Figure 10 I Photoresponsive characteristics of a bilayer MoS_2 photodetector without a top-gate electrode in the image sensor array. *I*–*V* curves of a photodetector without a top-gate electrode based on a bilayer MoS_2 channel under **a**, red **b**, green and **c**, blue light illumination with various incident power densities (λ_{ex} = 638 nm (R), 532 nm (G), 405 nm (B), and P_{inc} = 0.1, 0.2, 0.4, 0.8, 1.6, 3.2 mW cm^{-2}). **d**, Photoresponsivity, **e**, specific detectivity, and **f**, photosensitivity of the MoS_2 photodetector without a top-gate electrode calculated from Supplementary Fig. 10a–c. **g–i**, Photoswitching characteristics of the MoS_2 phototransistor under temporal light illumination with λ_{ex} = 638, 532, and 405 nm, respectively. All the switching curves were measured at V_{ds} = 5 V and P_{inc} = 4.5 mW cm^{-2} with the illumination frequency of 1 Hz. The rise and fall times were calculated as the times taken for the current to change from 20–80% and 80–20% of the maximum current, respectively.

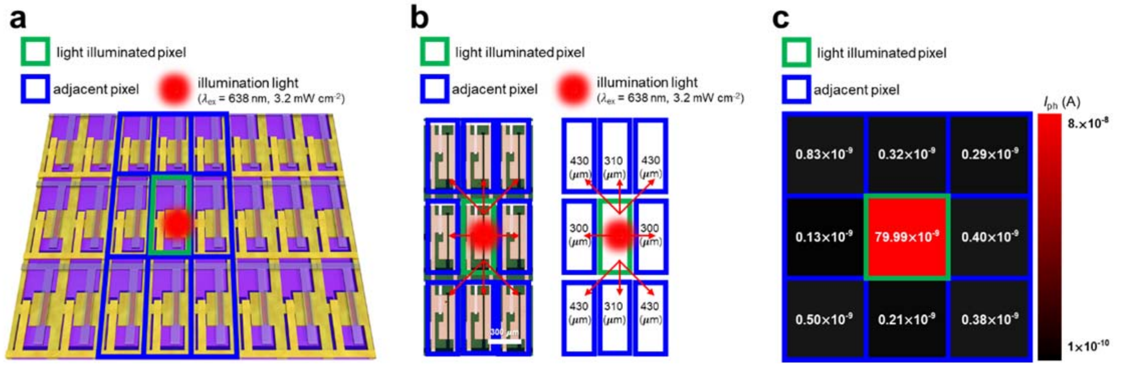

Supplementary Figure 11 l Cross-talk characterization of a light-illuminated pixel and its adjacent pixels. **a**, Schematic of the light-illuminated pixel and adjacent pixels. **b**, Optical microscopy image of the light-illuminated pixel and adjacent pixels. The specific length between channel of phototransistors in the light-illuminated pixel and that of phototransistors in adjacent pixels (top, bottom: 310 μm, right, left: 300 μm, and diagonal: 430 μm, respectively). **c**, Photocurrent mapping of the light-illuminated pixel and adjacent pixels under red light (λ_{ex} = 638 nm) with P_{inc} of 3.2 mW cm^{-2} at V_{gs} = -35 V and V_{ds} = 1 V.

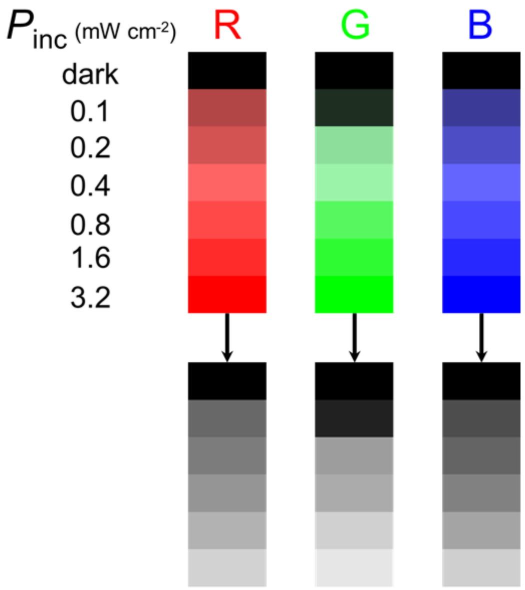

Supplementary Figure 12 I Grayscale image of a phototransistor in the image sensor array under various incident power density. The RGB color scale split by various incident power density and their grayscale equivalences, respectively.

Supplementary Table 1 l Comparison of the performance of the proposed photodetector with those of previous studies

Method	Layer	Large area	Spectrum range (λ)	Photo responsivity	Configuration	Active pixel array	Year	Ref.
CVD	1L	×	532 nm	780 A W^{-1} in ambient air	Phototransistor SiO_2 - Si	×	2013	S1
Two-step (thermolysis process)	3L	○	532 nm	0.57 A W^{-1}	MSM	×	2013	S2
CVD	1L	×	488 nm<br>514.5 nm	1.1 mA W^{-1}	MSM	×	2014	S3
CVD	1L	○	650 nm	10^7 A W^{-1}	Phototransistor SiO_2 - Si	×	2014	S4
CVD	1L	×	475 nm<br>535 nm<br>575 nm<br>630 nm	7.7 mA W^{-1}	MSM	×	2015	S5
CVD	2L	○	650 nm	32 mA W^{-1}	Phototransistor SiO_2 - Si	×	2016	S6
CVD	6L	○	515 nm	4–5 A W^{-1}	Phototransistor Al_2O_3 - Ti/Au	×	2017	S7
CVD	Multilayer	○	405 nm<br>532 nm	12.1 mA W^{-1}	MSM	×	2017	S8
CVD	1L	×	575 nm	308 mA W^{-1}	Homojunction photodiode	×	2017	S9
CVD	1L	○	405 nm<br>520 nm<br>658 nm<br>780 nm	20 mA W^{-1}	Phototransistor PVP - PEDOT:PSS	×	2017	S10
CVD	1L	×	632 nm	15.6 A W^{-1}	Phototransistor SiO_2 - Si	×	2017	S11
E-gun evaporator/CVD	2L	○	408 nm<br>515 nm<br>640 nm	8.0 mA W^{-1}	MSM	×	2019	S12
Pulsed laser deposition (PLD)	1–5L	○	300 nm<br>~<br>800 nm	1.96 A W^{-1}	MSM	×	2019	S13
Sputtering/ e-beam irradiation/ CVD	Multilayer	○	405 nm	3.7 A W^{-1}	Phototransistor SiO_2 - Si	×	2020	S14
MOCVD	1L	○	532 nm	150 A W^{-1}	Phototransistor SiO_2 - Si	○	2020	S15
Two-step (sputtering/CVD)	2L	○	405 nm<br>532 nm<br>638 nm	119.16 A W^{-1}<br>116.70 A W^{-1}<br>109.00 A W^{-1}	Phototransistor Al_2O_3 - IZO	○	2021	This work

Supplementary Table 2 l Comparison with the response time of the phototransistor in image sensor array without and with gate pulse.

	τ_r	τ_f	Gate pulse (V, ms)
Bilayer MoS_2 phototransistor	169.19 ms	104.8 ms	NA
	111.11 ms	22.99 ms	20, 40

Supplementary Note 1 | Simulations

The 2-D numerical simulations under illumination and in the dark are performed with a commercial software[S16]. Carrier transport was treated with the drift-diffusion formalism (see Supplementary Table 3 for the equations set). With this approach Poisson equation and continuity equations are solved self-consistently for each applied bias (in terms of gate-to-source and drain-to-source voltage) to obtain the electrostatic potential and the carriers' concentration. The light source is considered to be ideal, i.e., providing constant generation rates, for simplicity.

Supplementary Table 3 | Drift-Diffusion Equations used to model electrostatics and carrier transport in the device simulations.

Poisson Equation	$\nabla \cdot (\varepsilon \nabla \psi) = -q(p - n + N_D - N_A)$
Continuity Equation	$\mp \nabla J_{n,p} = q(G_{n,p} - R_{n,p})$
Drift-Diffusion Equations	$J_n = \mu_n n(-\nabla \psi) + D_n \nabla n$<br>$J_p = \mu_p p(-\nabla \psi) - D_p \nabla p$
Recombination Equation	$R_{n,p} = \dfrac{np - n_i^2}{\tau_n(p + p_1) + \tau_p(n + n_1)}$

The device structure implemented in the simulator is schematically represented in Fig. 1, with indication of device dimensions. The geometrical parameters adopted in the simulations are collected in Supplementary Table 4.

Supplementary Table 4 I Geometrical dimensions adopted in the device simulations.

**Symbol**	**Description**	**Parameter value**
L_G	Gate Length	15 µm
W_G	Gate Width	250 µm
t_{ox}	Gate Oxide Thickness	80 nm
t_{chan}	MoS_2 Channel Thickness	2 nm
t_{box}	Buried Oxide Thickness	300 nm

Supplementary Table 5 collects the MoS_2 material parameters used in the simulations. Supplementary Table 6 includes additional device parameters used in the simulations. Schottky barriers are considered at the boundaries of the MoS_2 layer with the source and drain contacts. SHR and Radiative recombination in the semiconductor layer are also taken into account. The Al_2O_3 is the gate oxide material ($\varepsilon_r \sim 7$).

Simulation results with no traps included are shown in Supplementary Figure 13a. No appreciable negative V_{th} shift can be observed in this case in contrast with the case with traps included, see Supplementary Figure 13b. This confirms the hypothesis concerning PG as the dominant effect determining photoresponsivity. Simulated band diagrams at different V_{gs} are shown in Supplementary Figure 14 under light illumination (i.e., $P_{inc} \approx 10^{-1}$ mW cm^{-2}) with (Supplementary Figures 14a-e) and without traps (Supplementary Figures 14f-j) included in the simulations. When light generates electron-hole pairs the excess holes get trapped into the trap level at 0.2 eV above E_V causing V_{th} to shift. This is illustrated by Supplementary Figure 15, that shows the trapped charge density (N_T^+) at different V_{gs} in the dark and with light illumination, clearly indicating that more charge gets trapped in the latter case than in the former.

Supplementary Table 5 l MoS_2 material parameters[S17].

Symbol	Description	Parameter value
E_G	Band Gap	1.4 eV
χ	Electron Affinity	4.4 eV
ε_r	MoS_2 Relative Dielectric Constant	5
N_C	Effective Density of States in the Conduction Band	1.12×10^{19} cm^{-3}
N_V	Effective Density of States in the Valence Band	2.54×10^{19} cm^{-3}
n_i	Intrinsic Carrier Concentration	2.93×10^{7} cm^{-3}
N_A	Doping Concentration (p-type)	5.0×10^{19} cm^{-3}
$\mu_{n,p}$	Carriers' Mobility	5 cm^2 V^{-1} s^{-1}
$\tau_{n,p}$	Carriers' Lifetime	1 μs

Supplementary Table 6 l Additional device parameters used in the simulations.

Symbol	Description	Parameter value
$\varepsilon_{r,ox}$	Relative Dielectric Constant (Al_2O_3)	7
Φ_G	Gate Contact Work-Function	5 eV (IZO)
$\Phi_{S,D}$	Source, Drain Contacts Work-Function	5 eV (Gold)

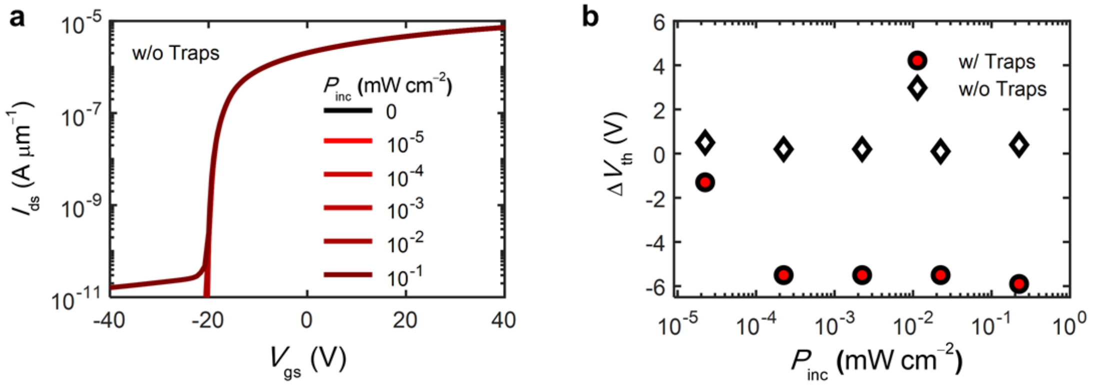

Supplementary Figure 13 I Additional simulation results. a, Simulated I_{ds}-V_{gs} with no traps included under light illumination, for different incident power densities (P_{inc}). **b,** Comparison of the Threshold Voltage Shift (ΔV_{th}) vs P_{inc} extracted from the I_{ds}-V_{gs} simulation with and without traps included.

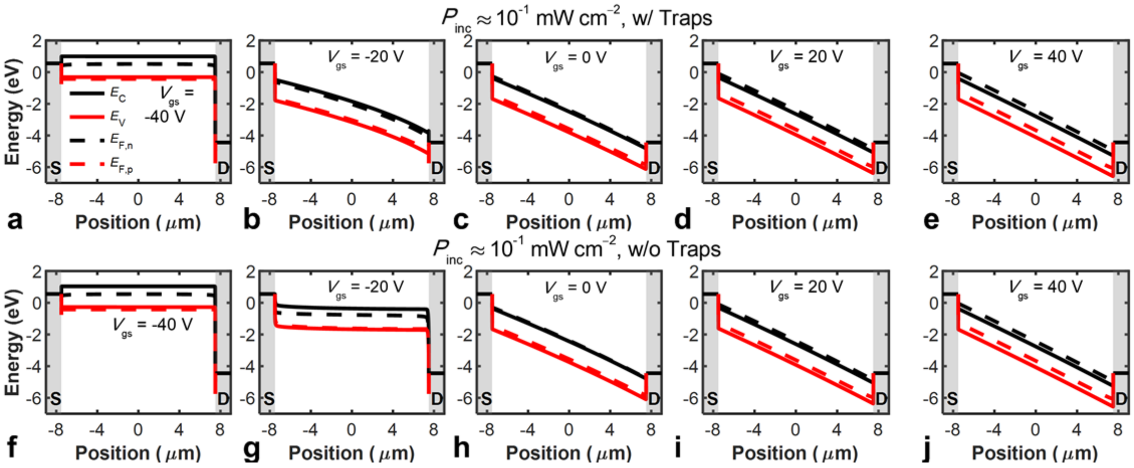

Supplementary Figure 14 | Simulated energy band diagrams for different V_{gs} = (-40, -20, 0, 20, 40) V under light illumination ($P_{inc} \approx 10^{-1}$ mW cm^{-2}) with (**a-e**) and without traps (**f-j**).

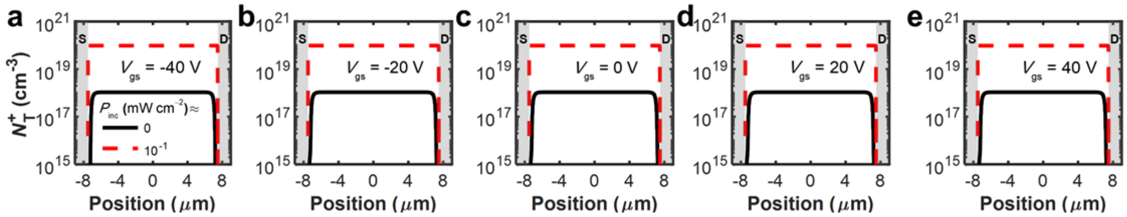

Supplementary Figure 15 l Trapped charge density. a-e, Trapped charge density (N_T^+) for different V_{gs} = (-40, -20, 0, 20, 40) V in the dark (P_{inc} = 0 mW cm^{-2}, black lines). and under light illumination ($P_{inc} \approx 10^{-1}$ mW cm^{-2}, red-dashed lines).

Supplementary References

S1. Zhang, W. et al. High□gain phototransistors based on a CVD MoS_2 monolayer. *Adv. Mater.* **25**, 3456-3461 (2013).

S2. Tsai, D.-S. et al. Few-layer MoS_2 with high broadband photogain and fast optical switching for use in harsh environments. *ACS Nano* **7**, 3905-3911 (2013).

S3. Perea-López, N. et al. CVD-grown monolayered MoS_2 as an effective photosensor operating at low-voltage. *2D Mater.* **1**, 011004 (2014).

S4. Zhang, W. *et al.* Ultrahigh-gain photodetectors based on atomically thin graphene-MoS_2 heterostructures. *Sci. Rep.* **4**, 1-8 (2014).

S5. Le, C. T. et al. Photosensitivity Study of Metal-Semiconductor-Metal Photodetector Based on Chemical Vapor Deposited Monolayer MoS_2. *J. Nanosci. Nanotechnol.* **15**, 8133-8138 (2015).

S6. Chen, C. et al. Large-scale synthesis of a uniform film of bilayer MoS_2 on graphene for 2D heterostructure phototransistors. *ACS Appl. Mater. Interfaces* **8**, 19004-19011 (2016).

S7. Choi, C. et al. Human eye-inspired soft optoelectronic device using high-density MoS_2-graphene curved image sensor array. *Nat. Commun.* **8**, 1-11 (2017).

S8. Lee, S. H. et al. Large□area growth of multi□layered MoS_2 for violet (～405 nm) photodetector applications. *Phys. Status Solidi A* **214**, 1700221 (2017).

S9. Zhang, X. et al. Poly(4-styrenesulfonate)-induced sulfur vacancy self-healing strategy for monolayer MoS_2 homojunction photodiode. *Nat. Commun.* **8**, 1-8 (2017).

S10. Kim, T.-Y. et al. Transparent large-area MoS_2 Phototransistors with inkjet-printed components on flexible platforms. *ACS Nano* **11**, 10273-10280 (2017).

S11. Nie, C. et al. Ultrafast growth of large-area monolayer MoS_2 film via gold foil assistant CVD for a highly sensitive photodetector. *Nanotechnology* **28**, 275203 (2017).

S12. Chen, S.-W. H. et al. Enhanced wavelength-selective photoresponsivity with a MoS_2 bilayer grown conformally on a patterned sapphire substrate. *J. Mater. Chem. C* **7**, 1622-1629 (2019).

S13. Jiao, L. et al. Layer-dependent photoresponse of 2D MoS_2 films prepared by pulsed laser deposition. *J. Mater. Chem. C* **7**, 2522-2529 (2019).

S14. Park, H. et al. Exceptionally Uniform and Scalable Multilayer MoS_2 Phototransistor Array Based on Large-Scale MoS_2 Grown by RF Sputtering, Electron Beam Irradiation, and Sulfurization. *ACS Appl. Mater. Interfaces* **12**, 20645-20652 (2020).

S15. Jang, H. *et al.* An atomically thin optoelectronic machine vision processor. *Adv. Mater.* **32**, 2002431 (2020).

S16. Synopsys Sentaurus SDevice Manual (O-2018.06) (2018).

S17. K.-C. Wang *et al.* Control of interlayer physics in 2H transition metal dichalcogenides. *J. Appl. Phys.* **122**, 224302 (2017).
